# Cutting-Edge Research in Sports Biomechanics: From Basic Science to Applied Technology

**DOI:** 10.3390/bioengineering10060668

**Published:** 2023-06-01

**Authors:** Wei-Hsun Tai, Rui Zhang, Liangliang Zhao

**Affiliations:** 1 Graduate School, Chengdu Sport University, Chengdu 610000, China; 2 School of Physical Education, Quanzhou Normal University, Quanzhou 362000, China; 3 Key Laboratory of Bionic Engineering (Ministry of Education, China), Jilin University, Changchun 130022, China; llzhao21@mails.jlu.edu.cn

## 1. Introduction

Sports biomechanics is the study of the mechanical principles of human movement and how they apply to sports performance [1]. It involves the analysis of motion, force, and energy during sports activities and aims to understand the biomechanical factors that influence performance and injury risk [2]. Sports biomechanics is an interdisciplinary field that combines elements of engineering, physics, anatomy, and physiology to help athletes optimize their performance and reduce the risk of injury [3]. Understanding the biomechanics of sports is important because it can provide athletes with insights into how to improve their technique and training methods and develop new training methods and equipment that can help them perform at their best [4]. In addition to helping athletes improve their performance, sports biomechanics can also play a critical role in reducing the risk of injury [5]. By understanding the biomechanical factors that contribute to sports injuries, such as overuse or poor technique, coaches and trainers can develop injury prevention strategies that are tailored to the specific needs of individual athletes [1,2,3,4,5].

This Special Issue contains 11 studies that present new knowledge in the fields of sports biomechanics and bionic engineering. Our aim is to encourage the dissemination of this new knowledge and provide guidance to potential authors who are interested in submitting their manuscripts to our bioengineering journal. For this Special Issue, the editors, editorial board members, and editorial staff have sought highly valued research that advances scientific knowledge and will have a positive impact on sports biomechanics and bionic applications in sports. The authors cover a wide variety of important, innovative, and timely topics in the field. The themes include sports technology analysis [6,7,8,9,10], the mechanics of human motion [11,12,13,14,15], bionic applications and equipment design [16], and the mechanisms of sports injuries [12,13]. In this editorial, we will discuss the current state of sports biomechanics and the direction it is headed.

As mentioned above, sports biomechanics is crucial to athletes’ success as it offers insights that allow athletes to optimize their performance, reduce the risk of injury, and develop new training methods and equipment [17,18,19]. Biomechanics and bionics have transformed the field by focusing on injury prevention and rehabilitation, developing personalized equipment, and utilizing computational modeling and artificial intelligence to optimize training regimens [20]. Continued investment in research is necessary to advance sports science further, develop new technologies and methodologies, and enhance athlete performance and safety. It is essential to support research in this area to ensure that the future generations of athletes can access the latest advancements in sports science and reach their full potential.

## 2. Application of Scientific Principles in Sports Biomechanics

Sports biomechanics is an interdisciplinary field that combines fundamental scientific principles with advanced technological tools to study the mechanics of human movement and its application in sports performance [1,2]. Basic scientific research in sports biomechanics involves the analysis of human movement, muscle and joint mechanics, neuromuscular control, the kinematics and kinetics of sports movements, and biomechanical modeling and simulation [3,4,5]. By understanding these biomechanical principles, researchers can identify the most efficient and effective techniques for athletes to use in their training and competition.

Applied technology is an essential component of sports biomechanics research, allowing the development and use of tools and equipment to measure and analyze human movement during sports activities [5]. Wearable sensors, motion capture systems, force plates, 3D printing, and virtual reality are just a few examples of applied technologies used in sports biomechanics. These tools provide precise measurements and data that are used to analyze and optimize human movement [6,7,8,9,10,11,12,13,14,15,16,17,18,19]. Furthermore, they enable the development of custom-fit equipment, training programs, and injury prevention strategies that are tailored to athletes’ individual needs.

The significance of sports biomechanics research lies in its ability to optimize sports performance while reducing the risk of injury [1,2,3,4,5]. Athletes and coaches can, thus, apply biomechanics to identify the most effective training methods and equipment to use with this goal in mind [21,22,23,24,25]. The integration of basic science and applied technology in sports biomechanics research has led to the development of new training methods, equipment, and injury prevention strategies and has contributed to a better understanding of the biomechanical response to sports activities.

In conclusion, sports biomechanics is an interdisciplinary field that combines fundamental scientific principles with advanced technological tools to study the mechanics of human movement during sports activities. Applied technology plays a crucial role in sports science research by enabling the development and utilization of tools and equipment to measure and analyze human movement during sports activities. Via continued research and development, the field of sports biomechanics has the potential to revolutionize the way athletes train and compete, leading to optimized performance and a reduced risk of injury.

## 3. Application of Bionic Engineering Technology to Sports

Bionics is an interdisciplinary field that draws inspiration from nature to design and optimize artificial systems and devices [26,27,28]. It combines biology, engineering, and materials science to imitate the structure, function, and movement of living organisms [29,30]. This innovative and forward-looking approach produces new technologies that integrate the empirical, theoretical, and practical knowledge of biological origins [29,31,32].

Modern sports have become extremely competitive, and athletes’ performance depends not only on their personal abilities and training but also on high-quality equipment and clothing to help them succeed in competitions [32]. Bionics has a wide range of research areas in sports, including biomimetic protective/assisted sportswear, biomimetic protective/assisted sports footwear, and biomimetic/assisted sports equipment. Among them, the application and development of sports footwear is the most in-depth research area [26,33]. In terms of biomimetic protective/assisted sports apparel, bionics mainly studies how to design and optimize sports apparel by imitating the materials and tissue structures of living organisms in nature to better adapt to the characteristics and needs of human movement [32].

The significance of bionics research lies in its ability to improve performance and the efficiency of sports equipment, reduce sports injuries, and enhance the sports experience. By conducting research in bionics, we can better understand the principles and adaptability of biological movement and apply them to the design of sports equipment, creating sports equipment that better fits human movement characteristics and needs [16,26,28,29,31]. The application of bionics in sports can have a profound impact beyond the field of athletics. It not only enhances athletes’ performance and provides new ideas for related disciplines but also promotes the development and innovation of sports equipment, ultimately improving enterprises’ competitiveness and market share. Additionally, bionics research can deepen our understanding of the mysteries of nature and life, driving the progress of science, technology, and human civilization.

In summary, the application of bionics covers various aspects of sports equipment, from design, materials, and structure to function and control, and has extensive and in-depth research. These applications not only improve performance and the user’s experience but also offer new ideas and methods for the research and promotion of sports equipment.

## 4. Future Perspectives

The integration of sports biomechanics and bionics has the potential to transform sports and athletics by optimizing performance, reducing the risk of injury, and providing personalized training recommendations. For example, biomechanical analyses can help identify and correct flawed movement patterns that may lead to injury [12,13,25]. Bionic technologies can also provide support and protection to the musculoskeletal system, reducing the risk of injury during training and competition. Furthermore, the integration of advanced technologies into sports equipment and clothing can provide athletes with real-time data on their performance, allowing for more precise training and competition strategies [16,32,33]. However, as with any new technology, ethical considerations must be taken into account to ensure fairness and equality among athletes. Overall, the integration of sports biomechanics and bionics has the potential to significantly enhance human physical capabilities and transform the way we approach sports and physical activity.

## 5. Conclusions

Cutting-edge research in sports biomechanics is advancing our understanding of human movement and improving sports performance. The research provides insights into the fundamental principles of human movement and how they apply to sports performance. Applied technologies are developing new tools and techniques for measuring and analyzing sports movements, while bionic technologies are pushing the boundaries of what is possible for human performance. Together, these areas of research are shaping the future of sports biomechanics and opening up new possibilities for athletes to reach their full potential.
